# Impact of Heated Tobacco Products, E-Cigarettes, and Cigarettes on Inflammation and Endothelial Dysfunction

**DOI:** 10.3390/ijms24119432

**Published:** 2023-05-29

**Authors:** Svenja Belkin, Julia Benthien, Paul Niklas Axt, Theresa Mohr, Kai Mortensen, Markus Weckmann, Daniel Drömann, Klaas Frederik Franzen

**Affiliations:** 1 Medical Clinic III, Site Lübeck, University Hospital Schleswig-Holstein, 23562 Luebeck, Germany; 2 Airway Research Center North (ARCN), German Center for Lung Research (DZL), 22927 Großhansdorf, Germany; 3 Cardiology Kiel, 24116 Kiel, Germany; 4 Clinic for Rhythmology, Campus Lübeck, University Hospital Schleswig-Holstein, 23562 Luebeck, Germany; 5 Section for Pulmonary Pediatrics, Campus Lübeck, University Hospital Schleswig-Holstein, 23562 Luebeck, Germany

**Keywords:** endothelial dysfunction, risk stratification, inflammation, smoking, heating, vaping, JUULing, JUUL, heated tobacco product, electronic cigarette

## Abstract

In addition to the market launch of heated tobacco products (HTPs) and the JUUL as well as the EVALI, they caused a widespread discussion on the risk reduction compared to a combustible cigarette. Furthermore, first data showed harmful effects on the cardiovascular system. We, therefore, conducted investigations including a control group with a nicotine-free liquid. Forty active smokers were studied in two different approaches during and after consuming an HTP, a cigarette, a JUUL, or a typical electronic cigarette with or without nicotine in a partly double-blinded randomised, cross-over trial. Inflammation, endothelial dysfunction, and blood samples (full blood count, ELISA, multiplex immunoassay) were analysed, and arterial stiffness was measured. In addition to the cigarette, an increase in the white blood cell count but also in proinflammatory cytokines was shown for the various nicotine delivery systems. These correlated with the parameters of arterial vascular stiffness as a clinical parameter of endothelial dysfunction. It can be shown that even a single consumption of the different nicotine delivery system or cigarette leads to a significant inflammatory reaction followed by endothelial dysfunction and increased arterial stiffness causing cardiovascular disease. Inflammation, endothelial dysfunction, and arterial stiffness should be addressed in long-term observational studies.

## 1. Introduction

Cigarette smoking is one of the most frequent preventable causes of illness and premature mortality [1,2,3]. Furthermore, there has been a significant increase in the prevalence in the context of the COVID-19 pandemic and the war in Ukraine [4]. Smoking primarily causes diseases of the respiratory system, cancer, and the cardiovascular system. About a third of those who died from cardiovascular disease were smokers [5]. While the harmful effects of the filter cigarette are known not only in vitro but also in vivo with long-term data, these long-term data are lacking in the field of nicotine delivery systems, such as e-cigarettes, JUUL, and heated tobacco products (HTPs) or electronic as well as combustible shisha. The JUUL is a pod-using e-cigarette, where the nicotine was present as a special salt and enjoyed great popularity, especially among US young people, until it was banned by the FDA [6,7]. In the study “Gesundheit in Deutschland aktuell” (GEDA 2014/2015-EHIS), the Robert Koch Institute (RKI) was able to show that 20.8% of women and 27.0% of men in the adult population in Germany smoke [8]. Through the influence of advertising, smoking is associated by many people with “coolness” and success, which has been reinforced by the introduction of the e-cigarette and has also increasingly appealed to younger people [9]. According to the “Deutsche Befragung zum Rauchverhalten” (DEBRA study), which is a two-month, representative oral survey on smoking and consumption behaviour [4], the number of participants using a tobacco heater increased from 1.8% to 5.1% by 2021 [4]. In this context, nicotine consumption has an economic as well as a social significance. Diseases and health impairments caused by nicotine use entail treatment and care costs of EUR 97.24 billion euros annually and can even lead to incapacity to work and early retirement [3,10].

The most frequent application is probably the inhalation of nicotine. Different possibilities and devices are distinguished. In this paper, the classic filter cigarette, the e-cigarette, the JUUL, and the IQOS, as a representative of HTPs, are compared. Both HTPs and e-cigarettes have been designed to release nicotine as an aerosol without the classic combustion and to make it available to the body via inhalation via the respiratory tract. In the HTPs made of specially prepared tobacco, the residual water is evaporated together with the nicotine. In the case of the e-cigarette, a liquid, which is a mixture of propylene glycol and/or glycerol as well as flavourings and possibly nicotine, is vapourised. In 2014, there were already more than 450 different e-cigarettes with now different generations and more than 8000 different liquids [11].

Endothelial dysfunction refers to a functional disorder of the endothelium. The endothelium synthesises various vasoactive substances, such as nitric oxide (NO), which is a potent vasodilator. The intake of pollutants from tobacco smoke leads to a reduced release of NO. Due to increased concentrations of free radicals, oxidised low-density lipoprotein (LDL) is formed with a direct vascular damaging effect [12,13]. The release of cytokines leads to an inflammatory reaction and a proliferation stimulus of the smooth muscles. The working group of Nabavizadeh et al. could prove an impairment of the endothelial function in rats after a single use of the IQOS. However, a direct transfer to humans is not possible from this [14]. In addition, increased blood concentrations of carbon monoxide (CO) in smokers promote vasoconstriction. Altogether, this cascade as well as inflammation and vascular inflammation causes the risk of atherosclerosis and plays a major role in the development of atherosclerosis, respectively. The cardiovascular risk due to arterial stiffness can be predicted with the parameters pulse wave velocity (PWV), augmentation index (AIx75), and other parameters of central hemodynamics. The parameters of arterial stiffness are considered surrogate parameters for cardiovascular events, because the vessels directly reflect the aspect of endothelial dysfunction, atherosclerosis, and inflammation [15,16,17,18]. To put it simply, arterial stiffness reflects biological age. PWV describes the running speed of the arterial pulse wave from the heart to the periphery, and the augmentation index describes the summation of the ejected pressure wave and reflected pressure wave over the arterial vascular system [19,20]. Especially in old age and with other factors, gender plays an increasing role, and the sexes differ in the development of arterial stiffness [21].

The humoral factors of the non-specific immune response include proinflammatory cytokines, such as interleukin-6 (IL-6), and acute phase proteins, such as C-reactive protein (CRP). Both are considered to be easily detectable inflammatory parameters during an infection. Scott et al. and Higham et al. were able to demonstrate an increased production of IL-6 after exposure to cigarette smoke and e-cigarette vapour, respectively [22,23,24]. The research group led by Sawa et al. was able to demonstrate both increased IL-6 plasma levels and an increase in oxidative stress after they applied IQOS aerosols in mice [25]. IL-6 as well as highly sensitive CRP are considered parameters for the prediction of cardiovascular risk [26,27]. However, there are some more cytocins which affect the inflammatory response caused by smoking or vaping. Ouyang et al. showed that cigarette smoke contains potent inhibitors of cytokine production such as interleukin-2 (IL-2), interferon (IFN), and tumour necrosis factor α (TNF α) c.f. [28]. Other examples include the anti-inflammatory interleukin-4 and interleukin-10 and the proinflammatory interleukin 8.

Inflammation and endothelial dysfunction are not stand-alone parameters but interact directly with each other and with arterial stiffness. Therefore, we investigated the question of the effect of acute consumption of combustible cigarettes, e-cigarettes, and tobacco heaters on inflammation and arterial vascular stiffness in experimental approaches.

## 2. Results

### 2.1. Baseline Characteristics

Baseline characteristics for all subjects are presented in Table 1 (first approach) and in Table 2 (second approach).

### 2.2. Cigarette, E-Cigarette, and HTPs Increased Leukocytes and Lymphocytes

**Figure 1:**
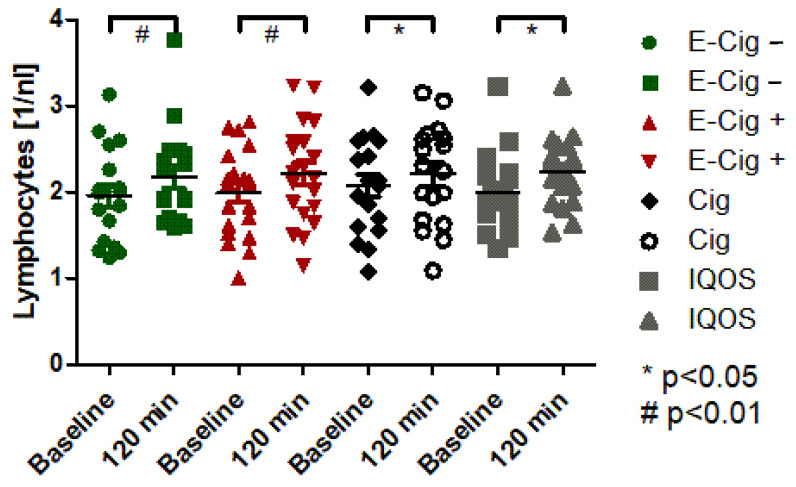
Lymphocytes in EDTA blood before and 120 min after application of the experimental condition. Green dots represent the E-cig–, red dots represent the E-cig+, black dots represent the cig, and grey dots represent the IQOS. Statistical significances are given for *p*-values of *p* < 0.01 with # and with * for 0.01 < *p* < 0.05.

The blood tests show that the paired *t*-test of the cigarette and IQOS shows a significant increase in the total leukocyte count (*p* < 0.05). Furthermore, the paired *t*-test for the e-cigarette without nicotine (*p* < 0.01), the e-cigarette with nicotine (*p* < 0.001), and the IQOS (*p* < 0.001) shows a highly significant increase in lymphocytes (Figure 1).

For the paired *t*-test of the cigarette, however, there was a significant increase (*p* < 0.05). For the paired *t*-test of the e-cigarette with nicotine-containing liquid, a significant increase in monocytes can be detected (*p* = 0.05). In addition, the paired *t*-test of the IQOS shows a highly significant increase in eosinophil granulocytes (*p* < 0.01) within the first approach. The ANOVA between the groups did not show any differences between the different devices at any time for the lymphocytes (*p* > 0.05) or leukocytes (*p* > 0.05).

Leukocytes increased significantly 120 min after the use of the nicotine delivery system (JUUL™ (*p* < 0.05), HTP (*p* < 0.01), and E-cig groups (*p* < 0.01)) in the second approach. The immature granulocytes increased significantly in the JUUL™ group (*p* < 0.05) and showed a trend without reaching the significance level in the Cig group (*p* = 0.058). A significant increase was measured in the HTP group (*p* < 0.01) as well as in the E-cig group (*p* < 0.05) for the neutrophil granulocytes.

### 2.3. Cigarette, E-Cigarette, and HTPs Increased Inflammation Values

In the hsCRP ELISA determination, there is a significant increase in the paired t-test of the cigarette (*p* < 0.05) and a highly significant increase in the paired *t*-tests of e-cigarette with and without nicotine as well as the IQOS (*p* < 0.01). The ANOVA between the groups showed a significant difference between the four groups after 120 min (*p* < 0.05).

**Figure 2:**
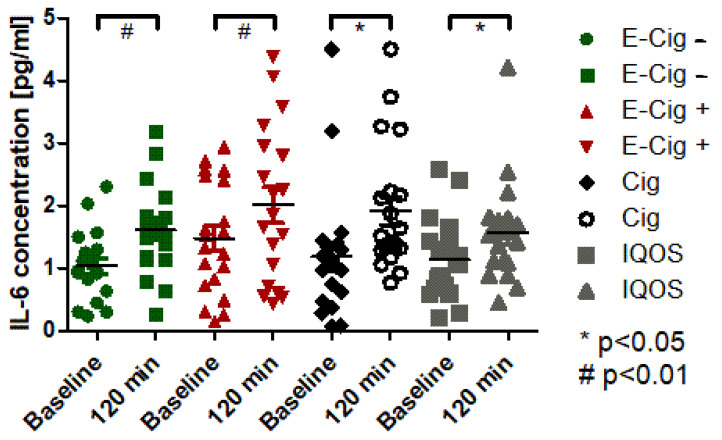
IL-6 concentration in plasma before and 120 min after application of the experimental condition. Green dots represent the E-cig–, red dots represent the E-cig+, black dots represent the cig, and grey dots represent the IQOS. Statistical significances are given for *p*-values of *p* < 0.01 with # and with * for 0.01 < *p*< 0.05.

In the evaluation of the IL-6 ELISA, a significant increase (*p* < 0.05) can be detected in the paired *t*-test of cigarette and IQOS, while a highly significant increase (*p* < 0.001) can be detected in the paired *t*-test of the e-cigarette with and without nicotine (Figure 2). The ANOVA between the groups did not show any differences between the different devices at any time for the IL-6 concentration (*p* > 0.05).

IL-6 determination by multiplex immunoassay, in contrast to ELISA determination, shows a significant increase for cigarette, e-cigarette (JUUL), and IQOS (*p* < 0.05) and a highly significant increase only for the e-cigarette without the nicotine-containing liquid (*p* < 0.001). The ANOVA between the groups showed a significant difference between the four groups after 120 min (*p* < 0.05).

The multiplex immunoassay showed a significant increase for IL-2 determination in the experimental arms of the e-cigarette (JUUL), IQOS, and nicotine-free e-cigarette (*p* < 0.05) and highly significant increase for the cigarette (*p* < 0.01). The corresponding determination of IL-8 describes a significant increase for the experimental arms of the e-cigarette (JUUL), IQOS, and e-cigarette with nicotine-free liquid (*p* < 0.05) and a highly significant increase for the experimental arm of the cigarette (*p* < 0.01). The multiplex immunoassay for TNF-α showed a significant increase for the e-cigarette (JUUL) (*p* < 0.05) and a highly significant increase for the cigarette, IQOS, and e-cigarette with nicotine-free liquid (*p* < 0.01). The ANOVA between the groups did not show any differences between the different devices at any time for the multiplex immunoassays (*p* > 0.05).

Determination of IL-4, CSF, IFN, and IL-10 failed to detect significant changes for any of the devices in the multiplex immunoassay.

### 2.4. Inflammation Due to Smoking and Vaping Causes Endothelial Dysfunction

**Figure 3:**
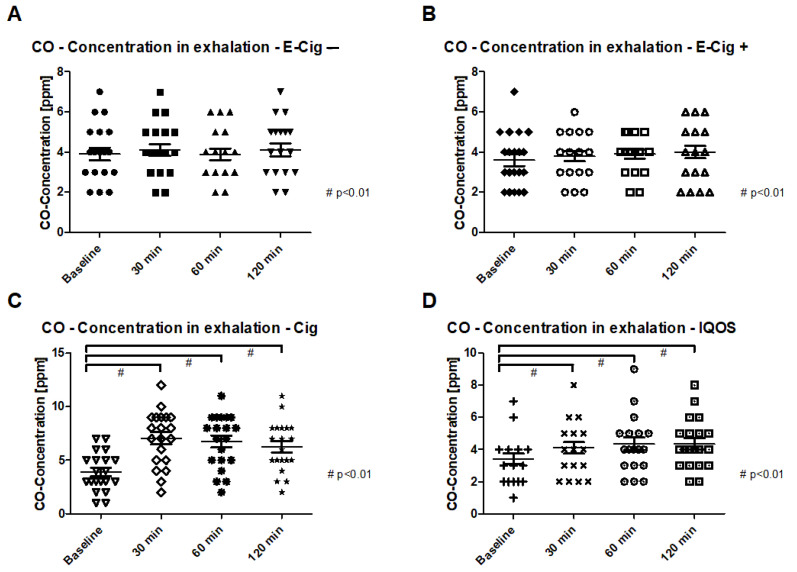
Carbon monoxide concentration (CO) in the exhalation with a point cloud and the mean value before and 120 min after application of the test condition. (**A**) represents the CO concentration for the E-Cig, (**B**) for the E-Cig+, (**C**) for the Cig, and (**D**) for the IQOS. Statistical significances are given for *p*-values of *p* < 0.01 with #.

In addition to the increase in the inflammation parameters in the blood samples examined, significant increases are also detectable in the CO breath measurement. In the paired *t*-test of the cigarette, a highly significant increase (*p* < 0.001) is detectable at all three measurement times. This high significance is also found in the paired *t*-test of the IQOS (*p* < 0.001) (Figure 3A–D). The ANOVA between the groups showed a significant difference between the four groups after 30 min, 60 min, and 120 min (*p* < 0.05).

### 2.5. Clinical Correlation of Inflammation, Endothelial Dysfunction, and Arterial Stiffness

**Figure 4:**
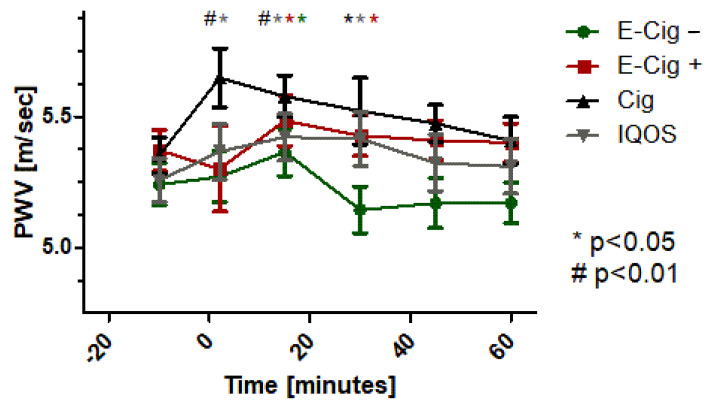
Pulse wave velocity (PWV) with values (mean ± SEM) before, directly after the use, and over time up to 60 min after application of the test condition. Green dots represent the E-cig–, red dots represent the E-cig+, black dots represent the cig, and grey dots represent the IQOS. Statistical significances are given for *p*-values of *p* < 0.01 with # and with * for 0.01 < *p* < 0.05. The colored asterisks represent the different test applications with their identical colors.

**Figure 5:**
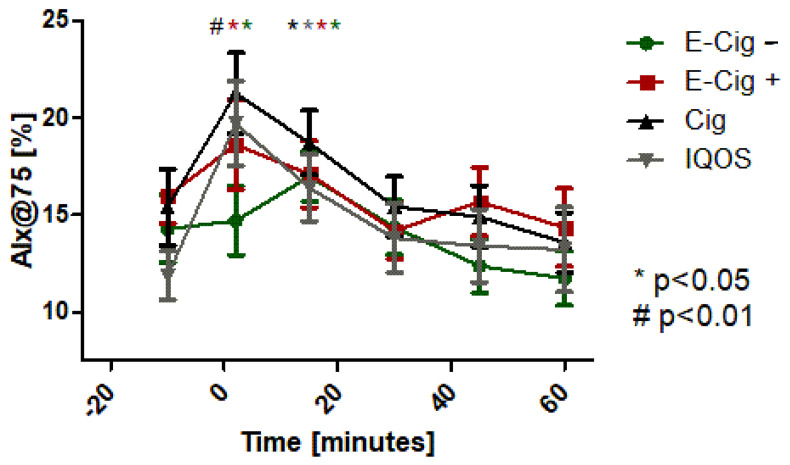
Augmentation index adjusted at heart rate of 75 bpm (AIx@75) with values (mean ± SEM) before, directly after, and over time up to 60 min after application of the test condition. Green dots represent the E-cig–, red dots represent the E-cig+, black dots represent the cig, and grey dots represent the IQOS. Statistical significances are given for *p*-values of *p* < 0.01 with # and with * for 0.01 < *p* < 0.05. The colored asterisks represent the different test applications with their identical colors.

Not only for the pulse wave velocity (PWV, Figure 4), as a surrogate parameter for cardiovascular events, but also for the augmentation index (AIx@75, Figure 5), significant negative changes, in the sense of an increase, could be statistically detected with the paired *t*-test after acute use of the nicotine delivery devices—IQOS (*p* < 0.05), e-cigarette without nicotine (*p* < 0.05), e-cigarette with nicotine (*p* < 0.05), or the combustible cigarette (*p* < 0.01). Gender differences could not be found in the statistical analyses for PWV or AIx@75. The ANOVA between the groups showed a significant difference between the four groups directly after the use of the devices for PWV and AIx@75 (*p* < 0.05). Further statistics showed a significant association in the context of a linear correlation between the increase and the increased inflammatory markers (hsCRP *p* < 0.05) and the cytokines IL-2 (*p* < 0.05), IL-6 (*p* < 0.05), IL-8 (*p* < 0.01), and TNF-alpha (*p* < 0.05).

## 3. Discussion

This paper shows one of the first studies of a head-to-head comparison in which all three devices, the filter cigarette, e-cigarette with nicotine-containing liquid, and heated tobacco product as well as the control arm of the e-cigarette with nicotine-free liquid, were examined within one study for inflammation, endothelial dysfunction, and arterial stiffness. In summary, we were able to show in our study not only an increase in the cellular immune response for all nicotine delivery devices and the combustible cigarette but also an increase in inflammatory markers and proinflammatory cytokines with a correlation to clinical data of arterial stiffness.

For the filter cigarette, path mechanisms for the risk of developing CVD have been shown in various studies so far. The smoke from a combustible cigarette led to an increase in markers of oxidative stress [29,30], inflammation [29], endothelial dysfunction [29,31], and elevated blood pressure levels [32] in both acute and prolonged use. Our findings are also in line with the results of Lee’s research group, who in a publication based on endothelial cells differentiated from induced pluripotent stem cells created a mechanistic model for the damage of the vapor of the e-cigarette via apoptosis, oxidative stress, and inflammation to endothelial dysfunction and consecutively the development of a cardiovascular disease [1].

Notably, our results showed changes in white blood cell count with an increase in leukocytes as well as monocytes. These findings are also in line with the findings for the combustible cigarette where the transient increase in white blood cells is a well-established cardiovascular risk factor [1,33]. The consumption of all four experimental arms leads to an increased lymphocyte count, albeit to different degrees. Because this also applies to e-cigarettes with nicotine-free liquid, the change is also nicotine-independent here. This underlines that even nicotine-free products cause a cellular immune response and the significance for harmfulness is thus more difficult to lack, even if the severity cannot be estimated in a real quantity based on cellular response. In addition, these results of white blood cell count now lead to the conclusion that there is an increased cardiovascular risk not only for the filter cigarette and e-cigarette regardless of the nicotine content in the liquid but also for the HTP.

Our results of the proinflammatory cytokines are also in line with the findings of Lee [1]. Based on the path mechanisms described by Lee et al. with regard to proinflammatory cytokines and their effect on endothelial dysfunction [1], we evaluate and interpret our results in the same direction. The working groups of Scott, Higham, and Sawa were also able to show connections between proinflammatory cytokines and the development of atherosclerosis, so that with the significant increase in the proinflammatory cytokines IL-2, IL-8, but also TNF-α and hsCRP for the various nicotine delivery systems or the combustible cigarette, damage to the vessel, in the sense of the aforementioned endothelial dysfunction, and a consecutively increased risk of developing CVD were observed [23].

The consumption of cigarettes and IQOS also leads to increased CO levels in the air we breathe. As described at the beginning, CO leads to an increase in vasoconstriction and thus also leads to endothelial dysfunction. However, a change in the endothelium is therefore not a separate process of CO or pro-inflammatory markers but is multifactorially conditioned.

Another unique feature of this work is the link between the laboratory results and the measurements of arterial vascular stiffness. This means that the effects of proinflammatory cytokines and white blood cell count as well as endothelial dysfunction based on them explain the acute changes in PWV and the augmentation index for the different nicotine delivery devices as well as the combustible cigarette.

Unfortunately, the investigative approach also has weaknesses. This includes, among other things, the very short observation period, so that only acute effects and no chronic effects can be observed, which should urgently be addressed in follow-up projects. In addition, the 20 test subjects constitute a rather small number of study participants. Due to the small number of subjects of each of the different experimental approaches, we have combined the two studies to consider the relationship between inflammation, arterial vascular stiffness, and endothelial dysfunction. Furthermore, we made supplementary laboratory tests and calculated additional statistics trying to add further explanations. A significant increase in the number of participants would lead to more valid measurement results. It is also not possible to verify with certainty whether all participants smoked or vapourised the respective devices exactly as specified. In addition, a correlation to the nicotine concentrations in the serum would be valuable in order to correlate the effects described in previous publications independently of the increasing serum-nicotine levels in this work.

## 4. Materials and Methods

### 4.1. Study Cohort and Design

The two different experimental approaches of single centre, four-arm semi-blind pilot studies included 20 different young, active smokers in each of the two approaches. The trial was designed as a crossover. The participants had no obligations between each visit regarding smoking or vaping. The participants were randomised by drawing pieces of paper out of a closed envelope for the order of the four different study arms on the first visit (Figure 1). The participants were recruited from students of the University of Luebeck. During the screening, all participants were checked for inclusion criteria: (i) smoking or vaping, (ii) no mental disorders, (iii) no cardiovascular diseases, (iv) no thyroid disease, (v) non-diabetic, (vi) no abnormalities in physical examinations, (vii) no hypertensive subject, or (viii) no hypercholesterolemia. Furthermore, only female participants using oral contraceptives were to be included in the study. All participants were asked to follow the guidelines. Therefore, the intake of alcohol and/or smoking cigarettes was prohibited 24 h prior to the measurement and a period of 48 h was required between each test day. The smoking-free time was tested by Micro + Smokerlyzer™ (Bedfont Scientific Ltd., Harrietsham, UK) with a cut-off of 6 ppm CO. Participants were excluded if previously enrolled in any other kind of study and if they declared as being strict non-smokers before they were given written informed consent. The studies had the permission of the local ethics committee by an amendment to a prior study with the topic of e-cigarettes and were registered with the DRKS (DRKS00012919).

**Figure 6:**
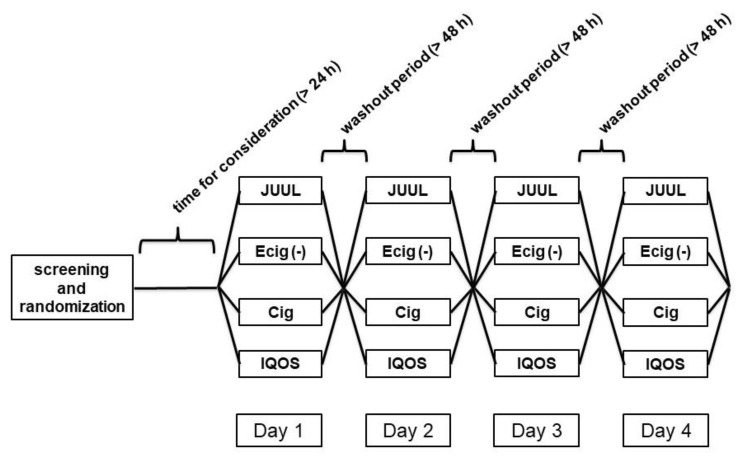
Flowchart of the study design.

The first approach consisted of four different study arms: (a) HTP 2.2 (Philip & Morris—New York, NY, USA; flavour “Bronze”, 0.5 mg nicotine per heet), (b) a commercial tobacco cigarette (Cig) (Marlboro Gold, Philip & Morris, New York, NY, USA; 0.5 mg nicotine), (c) e-cigarette with nicotine (ECig (+)) (DIPSE, Oldenburg, Germany, eGo-T CE4 vapouriser (third generation), 3.3 volts, 1.5 ohms, and 7.26 watts; 20 mg/mL, tobacco flavour), and (d) e-cigarette without nicotine (ECig (−)) (DIPSE, Oldenburg, Germany, eGo-T CE4 vapouriser (third generation), 3.3 volts, 1.5 ohms, and 7.26 watts; 0 mg/mL, tobacco flavour). Arms (c) and (d) were blinded by a person not involved in the investigators’ team. Therefore, both the investigators and participants were unaware of the kind of liquid used. The second approach also consisted of four different study arms: (a) HTP 2.2 (Philip & Morris, New York, USA; flavour “Bronze”, 0.5 mg nicotine per heet), (b) a commercial tobacco cigarette (Cig) (Marlboro Gold, Philip & Morris, New York, NY, USA,; 0.5 mg nicotine), (c) a JUUL™ e-cigarette (JUUL, San Francisco, USA) with the first-generation technology equipped with a JUUL™ pod of the flavour “Virginia Tobacco” (JUUL group, San Francisco, USA; 20 mg/mL nicotine), and (d) e-cigarette without nicotine (ECig (−)) (0 mg/mL, tobacco flavour). In the second experimental approach, blinding was not possible due to the two different types of e-cigarettes. As an example, the flowchart in Figure 6 is shown for the second approach of the study.

Additionally, neither the investigator nor the probands had any influence on the experimental sequence in both approaches. The use of HTPs was explained to the participants following the instructions made by an IQOS store. Furthermore, the participants were asked to smoke the cigarette as normal. During the study, smokers who were inexperienced in the use of e-cigarettes or HTPs were trained using e-cigarettes by an experienced e-cigarette user. All of them had to vape the e-cigarette with a minimum of a puff every 30 s for 10 puffs, in accordance with other publications [14]. Every puff had to last for 4 s in this publication. Each participant had to fulfil all four conditions to complete the study and to be analysed.

### 4.2. Measurement of Inflammation Markers, Endothelial Dysfunction, and Arterial Stiffness

For the blood tests, blood was taken from the test persons at two points in time: once at the beginning of the measurements directly before the consumption of the respective device (T0) and 120 min after the consumption of the device (T120). Blood was taken via a peripheral venous catheter inserted at the beginning of the respective series of measurements. The blood samples for determining a large blood count were sent to the clinic’s own laboratory for further analysis. Further blood samples were prepared for the ELISA tests and multiplex analysis. The Quantikine^®^ ELISA from R&D SYSTEMS^®^ (R&D Systems, Minneapolis, MN, USA), a bio-techne^®^ brand, was used for the highly sensitive determination of the Human C-reactive protein/CRP and for the determination of the Human IL-6 immunoassays. Additionally, the Bio-Plex^®^ Multiplex Immunoassay System from BIO-RAD (BIO-RAD Laboratories, Hercules/California, USA) was used for the sensitive determination of IL-2, IL-4, IL-6, IL-8, IL-10, CSF, INF, and TNF α. The tests were carried out in the institute’s own laboratory according to the manufacturer’s instructions.

The Micro + Smokerlyzer^®^ was used to measure CO in the breath before device consumption (T0), 30 min after (T30), 60 min after (T60), and after 120 min (T120).

The measurement of the parameters of arterial stiffness and peripheral as well as central hemodynamics was conducted by the Mobil-O-Graph^®^ (I.E.M, Stollberg, Germany). The Mobil-O-Graph operates with the oscillometric measuring technique and it determines the parameters of arterial stiffness, pulse wave velocity (PWV), augmentation index (AIx), and pulse pressure (PP). The AIx is given in this work as AIx@75 (augmentation index at a heart rate of 75 beats per minute). It determines the parameters of arterial stiffness, pulse wave velocity (PWV), augmentation index (AIx), and pulse pressure (PP). The AIx is given in this work as AIx@75 (augmentation index at a heart rate of 75 beats per minute). Therefore, a standard blood pressure cuff is placed over the right or the left arteria brachialis [34]. The ARCSolver transfer function processes the brachial waveforms, which are recorded at the level of diastolic blood pressure with the arm cuff, to the central systolic blood pressure. The recorded central waveforms facilitate the pulse waveform analysis. Thereby, the augmentation index and augmentation pressure are calculated [35]. The device had been programmed in advance to automatically start a new measurement every five minutes. The series of measurements was started 30 min before consumption of the respective devices and continued until 120 min after consumption. For validity verification, blood pressure was recorded on the other arm using the Omron MIT Elite Plus™ (Omron, Kyoto, Japan).

### 4.3. Statistical Analysis

Case number estimation was conducted via G-Power. The statistical analyses were carried out with the statistical software SPSS (SPSS 23 Inc., Chicago, IL, USA). Graphs were processed using GraphPad Prism (GraphPad Prism 5, San Diego, CA, USA). For statistical references, baseline mean values were taken and analysed for normal distribution by Kolmogorov–Smirnov tests before further analyses. In addition, data were analysed using paired Student’s *t*-tests and Wilcoxon tests, where appropriate, to compare individual time points within the four test settings within each single approach. ANOVA was used to analyse the differences between the four arms at the different time points. Due to the repetitive measurements, an ANOVA for repetitive measurement was used to analyse the interaction between time and the type of instrument used. Accordingly, post hoc tests (Bonferroni) were conducted via SPSS when an interaction occurred. For the calculation of the correlation between arterial vascular stiffness and the laboratory parameters, both experimental approaches were combined and the JUUL with the e-cigarette with nicotine was considered as one group. A linear regression was calculated as a statistical test procedure.

Unless otherwise stated, all data were expressed as mean and standard deviation (SD). A *p*-value between 0.05 and 0.075 was referred to as a trend without reaching a significance level. A *p*-value less than 0.05 was considered statistically significant and a *p*-value less than 0.01 was considered highly statistically significant.

## 5. Conclusions

In summary, the complementary studies of white blood cell count and proinflammatory cytokines provide an explanation for the clinical elevated parameters of arterial vascular stiffness as a correlate for endothelial dysfunction. This endothelial dysfunction could be shown not only for the e-cigarette, but also for the JUUL and heated tobacco products and are also nicotine-independent, so that an increased cardiovascular risk for the corresponding consumption can be postulated.

## Figures and Tables

**Table 1:** Baseline characteristics for all analysed 20 participants of the first approach.

Sex	All (n = 20)	Male (n = 10)	Female (n = 10)	*p*-Value
Age (years)	21.9 ± 2.6	21.5 ± 2.3	22.2 ± 2.9	0.79
Weight (kg)	72.3 ± 12.1	79.6 ± 11.0	64.9 ± 8.3	0.0109
Height (m)	1.76 ± 0.1	1.84 ± 0.1	1.68 ± 0.1	0.0099
BMI (kg/m^2^)	23.3 ± 1.9	23.5 ± 1.5	23.1 ± 2.2	0.8132
Waist (m)	0.795 ± 0.08	0.839 ± 0.067	0.751 ± 0.068	0.0354
Hip (m)	0.971 ± 0.07	0.99 ± 0.058	0.951 ± 0.0.8	0.5452
Cigarettes per day	1.9 ± 0.8	2.6 ± 0.7	1.6 ± 0.4	0.3228
Fagerström Test for Nicotine Dependence (points)	1.1 ± 0.4	0.9 ± 0.3	0.2 ± 0.1	0.1459

**Table 2:** Baseline characteristics for all analysed 20 participants of the second approach.

Sex	All (n = 20)	Male (n = 10)	Female (n = 10)	*p*-Value
Age (years)	25.2 ± 0.9	25.6 ± 0.4	24.7 ± 0.3	0.6063
Weight (kg)	78.2 ± 3.5	85.9 ± 1.9	68.8 ± 0.8	0.0104
Height (m)	1.76 ± 0.02	1.8 ± 0.0	1.7 ± 0.0	0.0040
BMI (kg/m^2^)	25.0 ± 0.8	25.9 ± 0.4	24.0 ± 0.2	0.2180
Waist (m)	0.821 ± 0.02	0.861 ± 0.07	0.771 ± 0.07	0.0181
Hip (m)	0.929 ± 0.02	0.935 ± 0.1	0.92 ± 0.07	0.7155
Cigarettes per day	2.1 ± 0.7	2.7 ± 0.1	1.2 ± 0.2	0.2832
Fagerström Test for Nicotine Dependence (points)	0.5 ± 0.2	0.8 ± 0.1	0.1 ± 0.0	0.1180

## Data Availability

Not applicable.
